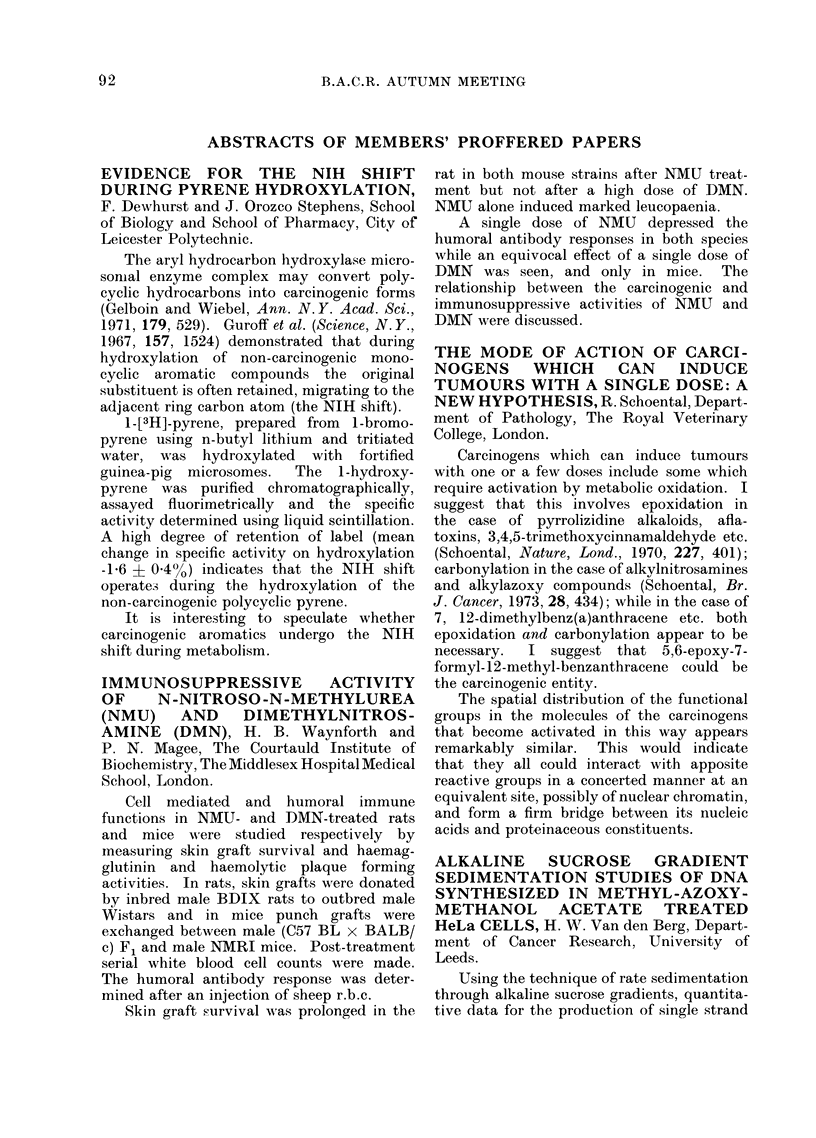# Proceedings: The mode of action of carcinogens which can induce tumours with a single dose: a new hypothesis.

**DOI:** 10.1038/bjc.1974.15

**Published:** 1974-01

**Authors:** R. Schoental


**THE MODE OF ACTION OF CARCINOGENS WHICH CAN INDUCE TUMOURS WITH A SINGLE DOSE: A NEW HYPOTHESIS,** R. Schoental, Department of Pathology, The Royal Veterinary College, London.

Carcinogens which can induce tumours with one or a few doses include some which require activation by metabolic oxidation. I suggest that this involves epoxidation in the case of pyrrolizidine alkaloids, aflatoxins, 3,4,5-trimethoxycinnamaldehyde etc. (Schoental, *Nature, Lond.*, 1970, **227**, 401); carbonylation in the case of alkylnitrosamines and alkylazoxy compounds (Schoental, *Br. J. Cancer*, 1973, **28**, 434); while in the case of 7, 12-dimethylbenz(a)anthracene etc. both epoxidation *and* carbonylation appear to be necessary. I suggest that 5,6-epoxy-7-formyl-12-methyl-benzanthracene could be the carcinogenic entity.

The spatial distribution of the functional groups in the molecules of the carcinogens that become activated in this way appears remarkably similar. This would indicate that they all could interact with apposite reactive groups in a concerted manner at an equivalent site, possibly of nuclear chromatin, and form a firm bridge between its nucleic acids and proteinaceous constituents.